# Risk Factors for Lymph Node Metastasis in Papillary Thyroid Carcinoma: A Systematic Review and Meta-Analysis

**DOI:** 10.3389/fendo.2020.00265

**Published:** 2020-05-15

**Authors:** Jingxin Mao, Qinghai Zhang, Haiyan Zhang, Ke Zheng, Rui Wang, Guoze Wang

**Affiliations:** ^1^School of Public Health, The Key Laboratory of Environmental Pollution Monitoring and Disease Control, Ministry of Education, Guizhou Medical University, Guiyang, China; ^2^College of Pharmaceutical Sciences, Southwest University, Chongqing, China; ^3^College of Food Science, Guizhou Medical University, Guiyang, China; ^4^Institute of Deep-Sea Science and Engineering, Chinese Academy of Science, Sanya, China; ^5^Department of Endocrine and Breast Surgery, The First Affiliated Hospital of Chongqing Medical University, Chongqing, China; ^6^Department of Pharmacology, College of Pharmacy, Guilin Medical University, Guilin, China

**Keywords:** papillary thyroid carcinoma (PTC), lymph node metastasis (LNM), risk factor, prognostic factor, meta-analysis

## Abstract

**Purpose:** To explore the risk factors that may predict the lymph node metastasis potential of these lesions and new prevention strategies in papillary thyroid carcinoma patients.

**Materials and Methods:** In total, 9,369 papillary thyroid carcinoma patients with 37.17% lymph node metastasis were analyzed (Revman 5.3 software) in this study. The PubMed and Embase databases were used for searching works systematically that were published through to January 22, 2020.

**Results:** Several factors were related to the increased risk of lymph node metastasis in patients with papillary thyroid carcinoma: age <45 years (pooled OR = 1.52, 95% CI = 1.14–2.01, *p* <0.00001); gender = male (pooled OR = 1.68, 95% CI = 1.51–1.87, *p* <0.00001); multifocality (pooled OR = 2.05, 95% CI = 1.45–2.89, *p* <0.0001); tumor size ≥1.0 cm (pooled OR = 3.53, 95% CI = 2.62–4.76, *p* <0.00001); tumor location at the upper pole 1/3 (pooled OR =1.46, 95% CI = 1.04–2.04, *p* = 0.03); capsular invasion + (pooled OR = 3.48, 95% CI = 1.69–7.54, *p* = 0.002); and extrathyroidal extension + (pooled OR = 2.03, 95% CI= 1.78–2.31, *p* <0.00001). However, tumor bilaterality (pooled OR = 0.85, 95% CI = 0.54–1.34, *p* = 0.49) and Hashimoto's thyroditis (pooled OR = 1.08, 95% CI = 0.79–1.49, *p* = 0.62) showed no correlation with lymph node metastasis in papillary thyroid carcinoma patients.

**Conclusion:** The systematic review and meta-analysis defined several significant risk factors of lymph node metastasis in papillary thyroid cancer patients: age (<45 years), gender (male), multifocality, tumor size (>1 cm), tumor location (1/3 upper), capsular invasion, and extra thyroidal extension. Bilateral tumors and Hashimoto's thyroiditis were unrelated to lymph node metastasis in patients with papillary thyroid cancer.

## Introduction

Thyroid carcinoma (TC) is the most frequent endocrine malignancy, accounting for approximately 3.8% of all newly diagnosed cancer (1). The incidence of TC has increased rapidly in recent 30 years with a female to male ratio of 3:1 (2, 3). Papillary thyroid carcinoma (PTC), medullary thyroid carcinoma (MTC), follicular thyroid carcinoma (FTC), and ana-plastic thyroid carcinoma (ATC) are the four main types of thyroid carcinoma (4). In addition, papillary thyroid microcarcinoma (PTMC) belongs to PTC. According to the histological classification of thyroid tumors by the World Health Organization (WHO), PTMC is defined as tumors with a maximum size of 10 mm or smaller (5). PTC is also the most familiar type of thyroid carcinoma, accounting for nearly 90% of all thyroid carcinomas with excellent prognoses (6). The general 10-year survival rate for middle-aged person with PTC is about from 80 to 95%, which is also related to an indolent clinical course (7).

Ultrasonography (US) and contrast-enhanced computerized tomography (CT) are commonly used but not especially accurate in clinical diagnosis of PTC, with low sensitivities of 38.9 and 27.5%, respectively (8). Following American Thyroid Association (ATA), fine-needle aspiration biopsy (FNAB) is considered to be the primary means of identifying benign and malignant nodules and selecting patients for surgery in clinical practice (9). In clinically diagnosis, the US or CT assistance during FNAB may enhance precision of cytological sampling and confirm nodal metastasis and may thus significantly reduce the false-negative diagnostic rate (10, 11).

Nowadays, the main treatment of primary/recurrent/advanced PTC is still reliant on surgical resection of total thyroidectomy (TT) (12). The radioiodine ablation (RAI) and lifelong levothyroxine therapy are commonly performed in intermediate- and high-risk patients (13). Although, PTC exhibit indolent behavior and bring a relatively low disease-specific mortality, with early-dissemination to local lymph nodes and oppression to organs, recurrence is comparatively common locally and distantly (14). In addition, the identification of risk factors of PTC is helpful for surgeons to evaluate the status of lymph nodes in PTC patients and determine whether preventive central lymph node dissection (CLND) is needed (15). Therefore, there is an urgent need for the identification risk factors that may predict the metastasis potential of these lesions and new prevention strategies. We conducted a systematic review and meta-analysis to assess the clinical characteristics of patients with PTC.

## Methods

### Search Strategy

The relevant published articles, including those of the PubMed and Embase databases, were used for identification up until January 22, 2020. The following keywords were used in searching: “risk factor OR predictive factor” AND “papillary thyroid carcinoma OR papillary thyroid microcarcinoma OR PTC OR PTMC.” Relevant articles were used to broaden the search scope, and all retrieved studies, reviews, and conference abstracts were retrieved by the computer. If multiple published studies describe the same population, we extracted only the most complete or recent one. Two authors (Jing-xin Mao and Qing-hai Zhang) independently completed the selection process and resolved the differences through discussion.

### Selection Criteria

The selection strategy used the several criteria: (a) prospective or retrospective original studies; (b) English language studies; (c) pathological confirmation of PTC during or after operation; and (d) available data on PTC risk factors and sufficient forms of data extraction to calculate the pooled OR.

Several exclusion criteria were adapted to exclude studies from meta-analysis: (a) reviews, case reports, editorials, letters to editors, meetings, or conference records; (b) studies included patients with thyroid cancer (e.g., FTC, MTC, or ATC) other than PTC; (c) insufficient data (e.g., <30 patients in the research); (d) research using big data (e.g., using SEER study data); (e) patients with a family history of thyroid cancer; and (f) studying period beyond 15 years.

### Data Extraction and Quality Evaluation

Two authors (Ke Zheng and Rui Wang) abstracted the following data from the included articles: first author, countries of study, years of publication, study design, study population (PTC or PTMC), number of cases, surgical intervention, and PTC-related risk factors. Age, gender, multifocal, tumor size, location, vascular invasion, thyroiditis (ETE), bilateral, and Hashimoto's thyroiditis (HT) were the risk factors of LNM in PTC patients. The Newcastle-Ottawa quality assessment scale was used to assess the quality of the research (16).

### Statistical Analysis

Statistical analysis of all meta analyses were performed using Ravman Manager version 5.3 (Cochrane Collaboration, Oxford, UK). The magnitude of the effect of each study was calculated by the odds ratio (OR) or the weighted mean difference (WMD) of the 95% confidence interval (CI) briefly. A *p*-value of <0.05 was considered statistically significant unless otherwise specified. In addition, the heterogeneity was quantified using the Q-test and the *I*^2^ statistic. When *p* > 0.1 and *I*^2^ <50%, a fixed-effect model was applied; otherwise, a random-effects model was used. The Begg funnel plot was used to test for possible publication bias.

## Results

**Figure 1:**
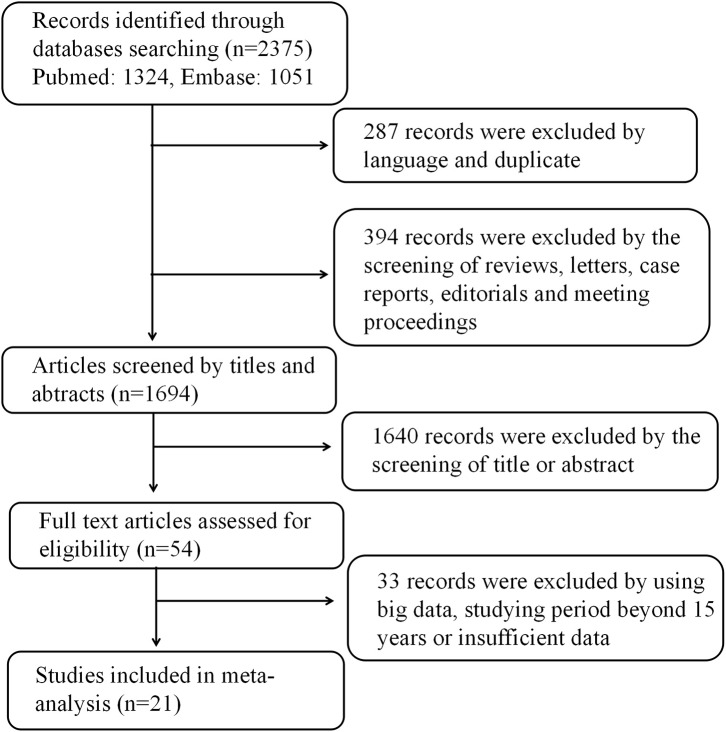
Flow chart of the study selection process.

After searching, a total of 2,375 studies were initially considered for inclusion in the meta-analysis. A total of 287 studies were excluded due to language and repetition. In addition, 394 studies were excluded in the form of reviews, case reports, editorials, letters to the editor, and summaries of conference or meeting proceedings. After investigating the titles and abstracts of the remaining 1,640 studies, a full review of 54 articles were evaluated. After a full review, a total of 21 studies that met our selection criteria were finally included in our meta-analysis. The selection flowchart of research is presented in Figure 1. The basic characteristics of the studies was included in Table 1. In all the risk factor analyses, no significant asymmetry was found in Begg's funnel plot.

**Table 1:** Basic characteristics of included studies.

**References**	**Country**	**Publication years**	**Study design**	**PTC/PTMC**	**Case number**	**Surgical intervention**	**Quality assessment**
An et al. (17)	China	2017	Retrospective study	PTC	146	TT/lobectomy + CND + LND	7
Chen et al. (18)	China	2015	Retrospective study	PTC	218	TT + bilateral CLND or ipsilateral and contralateral CLND	8
Hu et al. (19)	China	2018	Retrospective study	PTC	783	TT + ipsilateral CLND or ipsilateral LLND	8
Jeong et al. (20)	Korea	2017	Retrospective study	PTMC	625	TT + ipsilateral or bilateral CLND	7
Jiang et al. (21)	China	2014	Retrospective study	PTC	916	TT + bilateral CLND or lobectomy plus isthmusectomy+ ipsilateral CLND	7
Lv et al. (22)	China	2018	Retrospective study	PTC	1,442	TT + lobectomy	7
Liu et al. (23)	China	2019	Retrospective study	PTC	966	TT + lobectomy plus isthmusectomy + ipsilateral or bilateral CLND	8
Mao et al. (24)	China	2013	Retrospective study	PTC	389	TT + ipsilateral or bilateral CLND	7
Miao et al. (25)	China	2013	Retrospective study	PTC	184	TT + bilateral CLND	8
Noda et al. (26)	Japan	2015	Retrospective study	PTC	246	TT + CLND ±LLND	6
Park et al. (27)	Korea	2014	Retrospective study	PTMC	193	TT + bilateral CLND or lobectomy plus isthmusectomy + ipsilateral CLND	7
Shin et al. (28)	Korea	2014	Retrospective study	PTC	588	TT + ipsilateral or bilateral CLND ± LLND	8
Siddiqui et al. (29)	American	2016	Retrospective study	PTMC	273	TT or lobectomy ± CLND ± LLND	7
Tao et al. (30)	China	2017	Retrospective study	PTMC	66	TT or lobectomy + CLND + LLND	8
Wang et al. (31)	China	2017	Retrospective study	PTMC	150	TT + bilateral CLND or lobectomy plus Isthmusectomy + ipsilateral CLND	7
Wei et al. (32)	China	2015	Retrospective study	PTC	332	TT/NTT + bilateral CLND	7
Xue et al. (33)	China	2015	Retrospective study	PTC	1,555	TT + CLND	9
Yang et al. (34)	China	2014	Retrospective study	PTMC	291	TT + bilateral CLND	9
Yu et al. (35)	China	2018	Retrospective study	PTC	829	TT + bilateral or unilateral CLND	7
Zeng et al. (36)	China	2014	Retrospective study	PTMC	141	TT + ipsilateral CLND + LLND	8
Zhang et al. (37)	China	2015	Retrospective study	PTMC	178	TT + bilateral CLND or unilateral lobectomy plus isthmusectomy + ipsilateral CLND	6

### Prevalence of LNM and Variables in PTC

The prevalence of LNM population was clinicopathological variable in each study, ranging from 13.94 to 63.72%. Overall, LNM was confirmed among 3,482 patients of totally 9,369 PTC patients in this systematic review and meta-analysis.

### Risk Factors of LNM in PTC Patients (Table 2)

**Table 2:** Risk factors for lymph node metastasis in PTC patients.

**Risk factor**	**Pooled OR**	**95% CI**	***P*-value**
Age (<45 years)	1.52	1.14–2.01	<0.00001
Gender (male)	1.68	1.51–1.87	<0.00001
Multifocality	2.05	1.45–2.89	<0.0001
Tumor size (>1 cm)	3.53	2.62–4.76	<0.00001
Tumor location	1.46	1.04–2.04	0.03
Tumor bilaterality	0.85	0.54–1.34	0.49
Capsular invasion	3.48	1.69–7.54	0.002
Extrathyroidal extension	2.03	1.78–2.31	<0.00001
Hashimoto's thyroditis	1.08	0.79–1.49	0.62

#### Age

**Figure 2:**
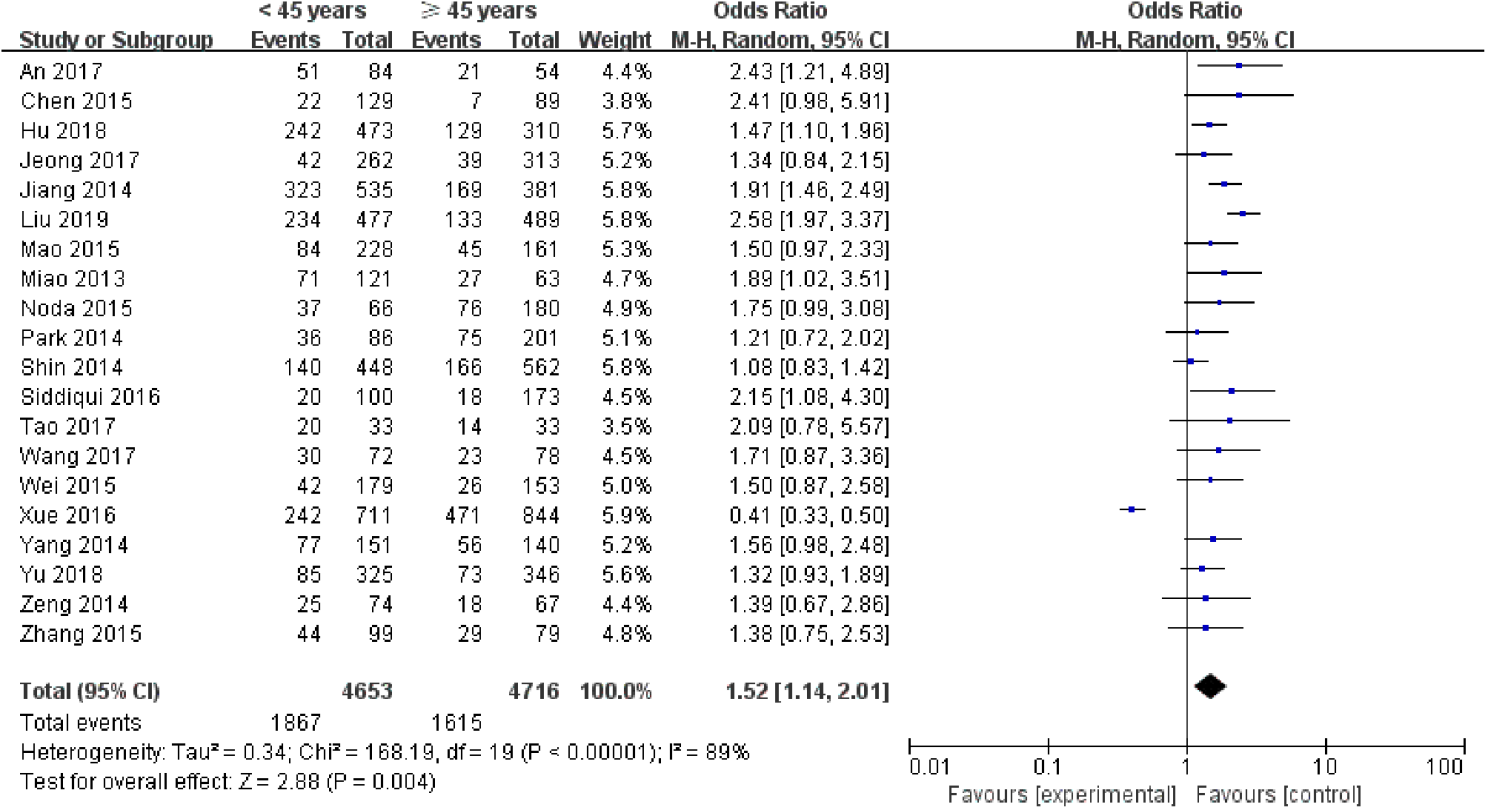
Forest plots of the association between age and PTC.

A random-effects model was utilized in the analysis (*p* = 0.004, *I*^2^ = 89%). Among patients with PTC, the rate of LNM was 40.12% in patients <45 years and 34.25% in the patients ≥45 years. The results indicated that age <45 years was related to an increased rate of LNM in PTC patients (pooled OR = 1.52, 95% CI = 1.14–2.01, *p* <0.00001) (Figure 2).

#### Gender

**Figure 3:**
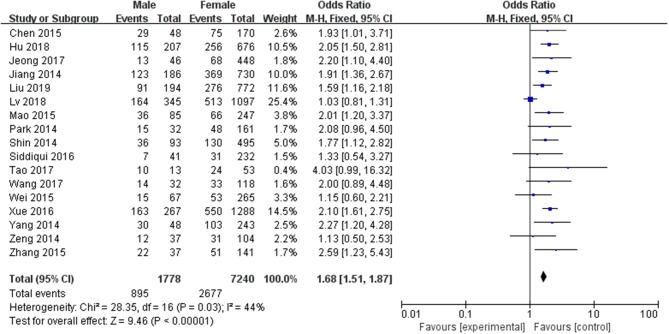
Forest plots of the association between gender and PTC.

A fixed-effects model was applied to analyze the data (*p* = 0.03, *I*^2^ = 44%). The prevalence of LNM in male PTC patients was significantly higher than that in female PTC patients (pooled OR = 1.68, 95% CI = 1.51–1.87, *p* <0.00001) (Figure 3).

#### Multifocality

**Figure 4:**
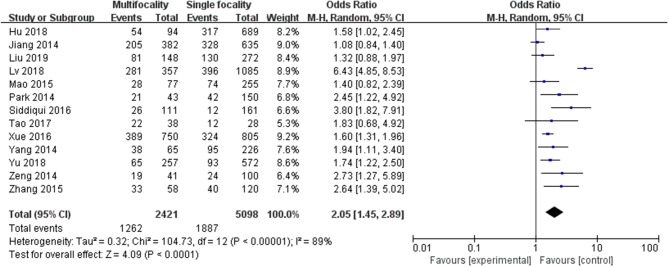
Forest plots of the association between multifocality and PTC.

A random-effects model was utilized in the analysis (*p* <0.00001, *I*^2^ = 89%). Thirteen included studies were evaluated. It was indicated that multifocality was significantly higher in association with LNM in PTC patients (pooled OR = 2.05, 95% CI = 1.45–2.89, *p* <0.0001) (Figure 4)

#### Tumor Size

**Figure 5:**
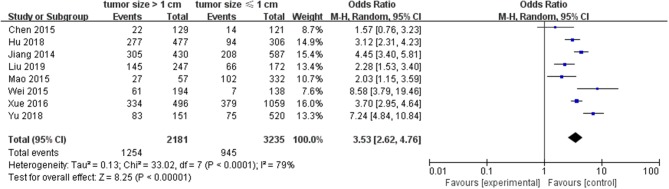
Forest plots of the association between tumor size and PTC.

A random-effects model was utilized to analyze the data (*p* <0.0001, *I*^2^ = 79%). Eight included studies were investigated. It was found that tumor size ≥1.0 cm was associated with a significantly higher LNM for PTC than tumors <1.0 cm (pooled OR = 3.53, 95% CI = 2.62–4.76, *p* <0.00001) (Figure 5).

#### Tumor Location

**Figure 6:**
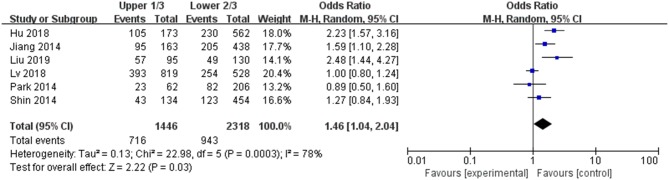
Forest plots of the association between location and PTC.

A random-effects model was applied in the analysis (*p* = 0.0003, *I*^2^ = 78%). Thyroid were divided into three areas including upper pole, middle pole, and lower pole. The upper pole 1/3 is divided into one category, and the middle and lower pole 2/3 are divided into one category. It was found that upper pole 1/3 was significantly associated with a high rate of LNM in PTC patients (pooled OR = 1.46, 95% CI = 1.04–2.04, *p* = 0.03) (Figure 6).

#### Tumor Bilaterality

**Figure 7:**
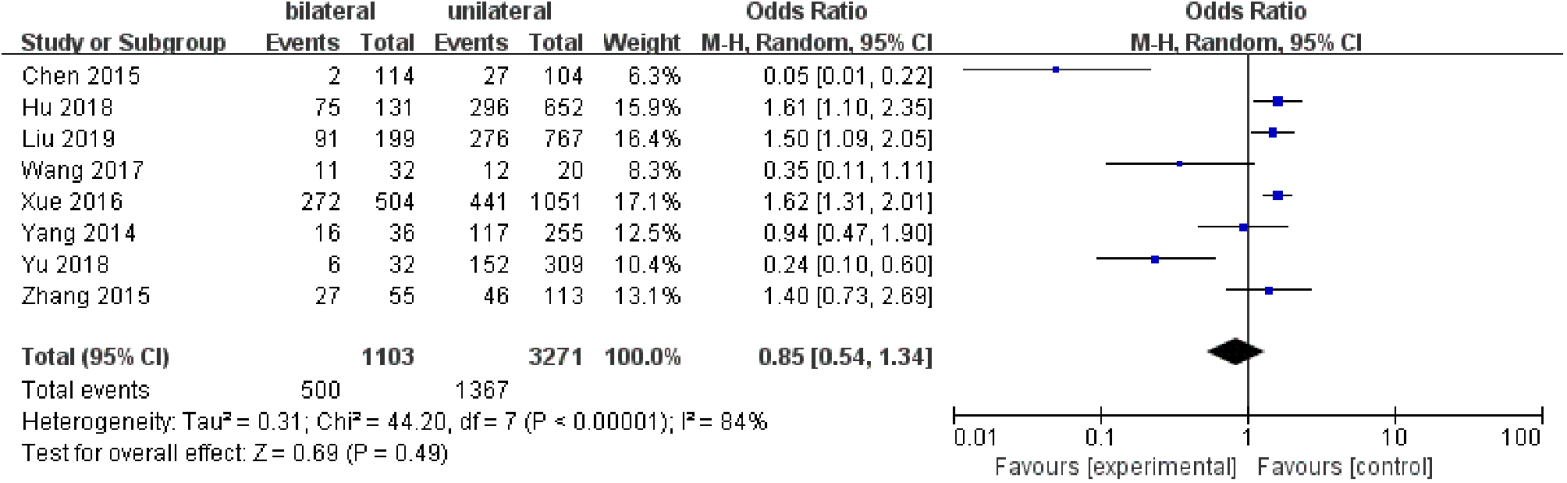
Forest plots of the association between bilateral tumors and PTC.

A fixed-effects model was utilized to analyze the data (*p* <0.00001, *I*^2^ = 84%). Eight included studies were evaluated for tumor bilaterality. It was found that both of unilateral tumors and bilateral tumors were not related to LNM in PTC patients (pooled OR = 0.85, 95% CI = 0.54–1.34, *p* = 0.49) (Figure 7).

#### Capsular Invasion

**Figure 8:**
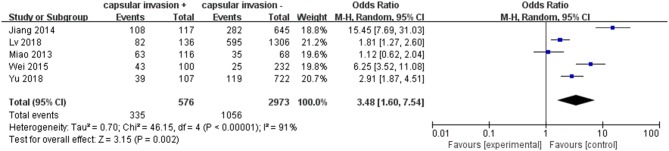
Forest plots of the association between capsular invasion and PTC.

A fixed-effects model was applied in the analysis involving capsular invasion (*p* <0.00001, *I*^2^ = 91%). Five included studies were investigated. Capsular invasion exhibited a relatively high odds ratio for LNM in PTC patients (pooled OR = 3.48, 95% CI = 1.69–7.54, *p* = 0.002) (Figure 8).

#### Extrathyroidal Extension

**Figure 9:**
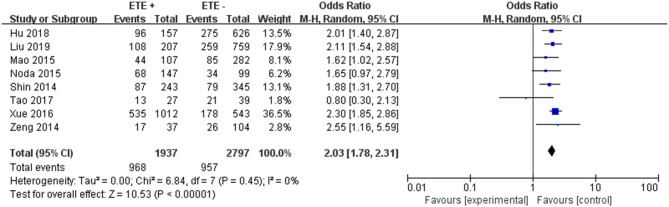
Forest plots of the association between ETE and PTC.

A random-effects model was used to analyze the data (*p* = 0.45, *I*^2^ = 0%). Eight included studies were investigated in this analysis. ETE was related to LNM in PTC patients (pooled OR = 2.03, 95% CI = 1.78–2.31, *p* <0.00001) (Figure 9).

#### Hashimoto's Thyroditis

**Figure 10:**
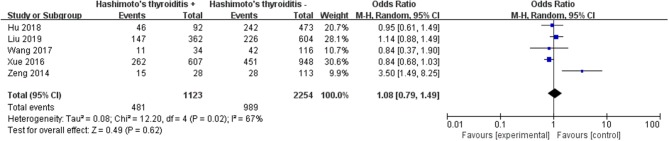
Forest plots of the association between HT and PTC.

A fixed-effects model was utilized in the analysis (*p* = 0.02, *I*^2^ = 67%). It was demonstrated that Hashimoto's thyroditis was not significantly related to LNM in PTC patients (pooled OR = 1.08, 95% CI = 0.79–1.49, *p* = 0.62) (Figure 10).

## Discussion

PTC derived from follicular cells is considered to be the most common malignant thyroid tumor, mostly occurring between 30 and 40 years old, and its 10-year survival rate is above 95% (38). However, PTC is also a common thyroid cancer that is considered to be the biological characteristic of metastasizing to the surrounding neck lymph nodes (39). In addition, lymph node metastasis has been reported as a major risk factor for recurrence in PTC patients who had regional at the time of diagnosis (40). According to the malignant results of preoperative US and FNA biopsy (e.g., whether lymph nodes metastasis was confirmed), the surgical treatment of patients was finally evaluated. Moreover, although PTC is considered to be a benign tumor that may have a good response to the treatment, some still develop recurrences that may be fatal (41). Therefore, it is necessary to continuously improve risk stratification system clinicopathological features of PTC that are associated with LNM.

A systematic review was conducted using Ravman Manager version 5.3 for systematic reviews and meta-analysis. In the meta-analysis we carried out, LNM was surveyed in 36.12% of patients with PTC. Patients with PTMC were also included in our review. In the present study, LNM was significantly related to the following clinicopathologic risk factors: age, gender, tumor size, tumor location, multifocality, capsular invasion, and ETE.

Age is a major prognostic factor for risk of LNM and recurrence in patients with PTC (42). Previous meta-analysis also demonstrated that age <45 years was related to the increased risk of LNM in PTC patients (43). In the present meta-analysis, it indicated that the patients age <45 years with PTC may have the increased risk of LNM in clinical practice (pooled OR = 1.52). Even though age ≥45 years is usually associated with a poor prognosis (LNM) and increased risk of recurrence, it was also reported that age <45 years is a poor predictor of prognosis of LNM in PTC patients (44). Therefore, whether an younger than 45 years old can be shown to be related to the increased risk of LNM in PTC patients still needs to be investigated in studies with a larger sample size.

Although the morbidity of thyroid cancer is relatively higher in women, the rates of PTC-induced malignancies and mortality are higher in men (45). In assessing patients with thyroid nodules, male sex is considered a risk factor for LNM, which may be suggestive of PTC (46). Based on the analysis result, we concluded that the gender of male was a significant risk factor for LNM in PTC patients (pooled OR = 1.68).

Among the clinical and pathological features that can be evaluated before and during surgery, tumor size is an important factor for tumor node metastasis (TNM) staging, and large tumors (tumor size >1 cm) are more vulnerable to aggression (47). In clinical diagnosis, a tumor size ≤ 1 cm represented microPTC/PTMC. According to the ATA guidelines, the PTMC exhibited less of a risk of LNM, and surgery for most PTMC was thus not recommended (9). However, it was also reported that LNM accounted for 64% of patients in the diagnosis of PTMC (48). Moreover, when LNM is found in PTMC patients, prophylactic central lymph node dissection (PCLND) is the standard treatment in clinical practice. In addition, LNM is also associated with higher risk of distant metastasis and about 11–22% risk of recurrence, especially for cervical lymph nodes (49). Therefore, TT combined with PCLND should be performed for PTMC patients presenting with LNM especially tumor size >1 cm (50). On account of our analysis data, PTC patients with tumor size >1 cm were at relatively higher risk of developing LNM than those with a tumor size ≤ 1 cm (pooled OR = 3.53). Previous research demonstrated that tumor size (>1 cm) is the best predictor of microcentric and lateral LNM, which was markedly affected lymph node recurrence in multivariate logistic analysis (51). Hence, careful lymph node dissection is strongly recommended for tumor size >1 cm in PTC patients even if it is considered as the preventive measure.

Multifocality is also considered an important risk factor for LNM in PTC patients. In addition, it was reported that the prognostic value of multifocality is particularly significant in PTC patients with tumor size >1 cm (52). Previous research has demonstrated that tumor multifocality is an independent risk factor of LNM in PTC patients after TT (53). Our finding was consistent with previous studies that the risk of LNM was higher in multifocality patients rather than single focality in PTC patients (pooled OR = 2.05). The result indicates that multifocality is an indicator of the aggressiveness of PTC tumors, showing a higher tendency for regional LNM. Therefore, multifocality may be associated with the state of disease progression, including risk stratification, management guidelines, and post-treatment monitoring in patients with PTC (54).

Six studies were analyzed for the correlation between tumor location and LNM in PTC patients. LNM was confirmed in 674 (46.74%) of 1,442 patients with upper 1/3 and in 985 (42.42%) of 2,322 patients with lower 2/3. According to our analysis data, the risk of LNM was conferred higher in patients with upper 1/3 than in those lower 2/3 (pooled OR = 1.46). In previous research, it was revealed that LNM was related to tumor location in upper 1/3 of the thyroid which is consistent with us (55). Nowadays, US combined with FNAB is usually used in the diagnosis of PTMC (tumor size ≤ 1 cm) patients by experienced doctors (10, 11). The tumor location of PTMC is one of the most important issues to determine whether active monitoring should be carried out. According to ATA guideline, an active surveillance approach as a management option is adopted for those low-risk PTMC patients (9). In clinical diagnosis, if the small tumor is located in the center of the thyroid lobe without LNM or thyroid capsule invasion, it can only be monitored by watching. Ito et al. published an article concluding that the characteristics that are not suitable for active surveillance included clinical LNM, distant metastasis, symptoms of recurrent laryngeal nerve or tracheal invasion, high-grade malignancy, and the presence of progression signs (i.e., gradually enlarged tumor size and the appearance of LNM) during active surveillance (56). In addition, it was also demonstrated that the 1/3 upper pole was the greatest independent factor that correlated with LNM in PTMC patients (57). Therefore, we suggest that, in cases with an upper 1/3 pole that are also invasive and include LNM, surgery should be performed; cases with a lower 2/3 pole that is non-invasive and without LNM should be monitored.

The relationship between tumor bilaterality and LNM in PTC patients was analyzed in eight studies. To reduce the risk of complications from PCLND and the potential to clear metastatic disease, bilateral central lymph node dissection (CLNM) rather than unilateral central lymph node dissection has been chosen for central neck dissection (58). In present study, there was no significant correlation (pooled OR = 0.85) between bilaterality and LNM in PTC patients.

Vascular invasion has been reported as a marker of an increased tendency toward hematogenic invasion and consequent increase in the relative percentage of LNM in patients with PTC which means a poorer prognosis ultimately (59). In addition, it was also reported that the presence of tumor capsular invasion does not adversely influence biological behavior (e.g., LNM) or survival of PTC (60). In our meta-analysis, it was found that capsular invasion was associated with LNM in PTC patients (pooled OR = 3.48).

In the presence of risk factors suggesting a possible increase in biological invasiveness, adequate postoperative treatment and close follow-up are essential. Tumor prognosis is related to the extent of extrathyroid expansion. The prognosis for patients with severely dilated extrathyroid disease is worse than patients with local microdilatation visible on histopathological examination (61). Previous research demonstrated that ETE have poorer prognosis including LNM than those without ETE in PTC patients (62). Our finding was consistent with previous studies that ETE was the increased risk of LNM in PTC patients (pooled OR = 2.03).

Previous studies suggest that the coexistence of HT is not related to LNM in PTC patients (63). However, it was also reported that there was a trend in patients with PTC and HT getting a better prognosis on which HT may have protected against central and lateral LNM (64). Our data demonstrated that there was no correlation between HT and LNM in PTC patients (pooled OR = 1.08).

According to the research, PTC is also considered to be a genetically driven disease. Therefore, it is necessary to understand the molecular mechanisms of the BRAF^V600E^ mutation and TERT promoter, which is reported upon in association with PTC. The activation mutation of serine threonine kinase v-RAF mouse sarcoma virus oncogene B1 (BRAF) is an important biomarker in human benign and malignant tumors, and most mutations affect BRAF^V600^ in exon 15 of BRAF gene (65). The BRAF^V600E^ mutation occurs in 30–80% PTC patients, which is the most common carcinogenic mutation (66). In addition, BRAF^V600E^ mutation is related to failure, recurrence, distant metastasis, and mortality in PTC treatment, which is considered an effective target for thyroid cancer (67). TERT is a catalytic subunit of telomerase that plays a dominant role in cell immortality and tumorigenesis (68). A mutation of TERT promoter was found in about 7.5% of PTCs, which induced the abnormal activation of telomerase is closely related to the invasive clinical behavior of papillary carcinoma (69). Moreover, it was revealed that TERT promoter mutation is a major indicator of extremely poor prognosis and aggressive clinicopathological characteristics (13). It also demonstrated that coexistence of BRAF^V600E^ and TERT promoter mutations are the most aggressive subgroup in PTCs patients, while PTCs with BRAF or TERT alone are less aggressive (70). Above all, to research those genetical mutations related to PTC may also help stratify patients into distinct risk groups and better assess patients' outcome.

Although the meta-analysis has explored several clinical and pathological predictors of LNM risk that may help surgeons choose appropriate treatment strategies in PTC patients, there are still some limitations that exist in our study. Firstly, there were only 21 studies that were included for predicting the risk of LNM in PTC patients. Secondly, the operation performed by different doctors may also have influence on the accuracy of data analysis, even following the standard mode and operation quality. Thirdly, most patients included in the study were from the same continent (Asia).

## Conclusion

Taken together, this meta-analysis investigated the following risk factors of LNM in PTC patients including age (<45 years), gender (male), multifocality, tumor size (>1 cm), tumor location (1/3 upper), capsular invasion, and ETE. Bilateral tumors and HT were not correlated with LNM in PTC patients. In addition, BRAF^V600E^ and TERT promoter mutations are also considered as the risk factors, which can help stratify PTC patients and better assess their prognosis.

## Data Availability Statement

The raw data supporting the conclusions of this article will be made available by the authors, without undue reservation, to any qualified researcher.

## Author Contributions

GW conceived and designed the project. JM, QZ, and HZ conducted the statistical analysis/meta-analysis and wrote the paper. KZ and RW abstracted the total data from the included articles. All authors contributed to manuscript revision, read, and approved the submitted version.

## Conflict of Interest

The authors declare that the research was conducted in the absence of any commercial or financial relationships that could be construed as a potential conflict of interest.

## Abbreviations

ATA: American Thyroid Association
ATC: Ana-plastic thyroid carcinoma
CLNM: Central lymph node metastasis
CND: Central neck dissection
CT: Computerized tomography
CI: Confidence index
ETE: Extra thyroidal extension
FNAB: Fine-needle aspiration biopsy
FTC: follicular thyroid carcinoma
HT: Hashimoto thyroiditis
LLND: Lateral lymph node dissection
LLNM: Lateral lymph node metastasis
LND: lymph node dissection
MTC: medullary thyroid carcinoma
NTT: Nearly total thyroidectomy
OR: Odd ratio
PTC: Papillary thyroid carcinoma
PTMC: Papillary thyroid microcarcinoma
PCLND: Prophylactic central lymph node dissection
RAI: Radioiodine ablation
SD: Standard deviation
TC: Thyroid carcinoma
TT: Total thyroidectomy
TNM: Tumor node metastasis
US: Ultrasound
WMD: Weighted mean difference
WHO: World Health Organization.
